# Caffeine is a risk factor for osteopenia of prematurity in preterm infants: a cohort study

**DOI:** 10.1186/s12887-017-0978-6

**Published:** 2018-01-22

**Authors:** Ebtihal Ali, Cheryl Rockman-Greenberg, Michael Moffatt, Michael Narvey, Martin Reed, Depeng Jiang

**Affiliations:** 1 Community Health Sciences Department, Faculty of Health Sciences, University of Manitoba, MS361K, 820 Sherbrook St, Winnipeg, MB R3A 1R9 Canada; 2 Department of Pediatrics and Child Health, Faculty of Health Sciences, University of Manitoba, Winnipeg, MB Canada; 3 Department of Radiology, Faculty of Health Sciences, University of Manitoba, Winnipeg, MB Canada; 4 Child Health Program, Winnipeg Regional Health Authority, Winnipeg, MB Canada

**Keywords:** Premature infants, Osteopenia of prematurity, Metabolic bone disease, Caffeine

## Abstract

**Background:**

Caffeine, the most commonly used medication in Neonatal Intensive Care Units, has calciuric and osteoclastogenic effects.

**Methods:**

To examine the association between the cumulative dose and duration of therapy of caffeine and osteopenia of prematurity, a retrospective cohort study was conducted including premature infants less than 31 weeks and birth weight less than 1500 g. Osteopenia of prematurity was evaluated using chest X-rays on a biweekly basis over 12 weeks of hospitalization.

**Results:**

The cohort included 109 infants. 51% had osteopenia of prematurity and 8% had spontaneous rib fractures. Using the generalized linear mixed model, caffeine dose and duration of caffeine therapy showed a strong association with osteopenia of prematurity. Steroids and vitamin D were also significantly correlated with osteopenia of prematurity while diuretic use did not show a statistically significant effect.

**Conclusion:**

The cumulative dose and duration of therapy of caffeine, as well as steroid are associated with osteopenia of prematurity in this cohort. Future studies are needed to confirm these findings and determine the lowest dose of caffeine needed to treat effectively apnea of prematurity.

## Background

Approximately 80% of bone mineralization of the newborn takes place during the third trimester of pregnancy because of the high rate of intrauterine growth [1]. Thus, preterm infants whom deprived of that period, are born with less bone mineral content. In addition, physiological adaptation of bone to extra-uterine life leads to an increase in bone resorption. This process occurs earlier in preterm than in term infants and can be accompanied by high risk of bone fragility and fractures [2]. Bone resorption appears to be more important than decreased bone formation in the pathogenesis of osteopenia of prematurity (OP) [3].

Almost 10% of infants are born prematurely worldwide, representing more than 15 million births every year. The incidence and severity of osteopenia of prematurity increase as the birth weight (BW) and gestational age (GA) decrease [4]. Preterm infants are known to have a lower bone density (BMD) and bone mineral content (BMC) [2] at the corrected age of term, as well as a lower weight and Ponderal index [5]. Moreover, preterm infants have lower bone strength at the distal tibia and radius compared to age and sex-matched controls, when assessed with computerized tomography as young adults [6].

In 1989, the incidence of OP was 55% of infants <1000 g and 23% of infants <1500 g at birth. A notable finding at this time was that OP risk showed an inverse relationship to lower GA and a direct relationship to duration of parenteral nutrition [7]. In 2009, a study reported pathological fractures in 30% of preterm infants with osteopenia [8].

Caffeine is the most commonly consumed pharmacologically active compound in the world [9]. In the neonatal intensive care units (NICU), it is one of the most commonly prescribed drugs to treat apnea of prematurity [10]. The half-life in neonates is 72–96 h (range: 40–230 h) and the time to peak serum concentration after oral administration ranges from 30 min to 2 h, whereas 86% of caffeine is excreted unchanged in urine [11]. The liver enzymes responsible for caffeine metabolism mature progressively with increasing GA. Girls were reported to have a higher rate of caffeine metabolism than boys [12]. Clearance of caffeine in infants born prematurely is markedly lower and the volume of distribution is higher than infants at term-equivalent age and beyond. Elimination of caffeine is initially depressed in extremely premature infants and then increases nonlinearly to final assessment at 6 weeks postnatal age [13]. It is well established that caffeine causes calciuria and creates negative calcium balance in preterm rats especially after prolonged use with compensatory increase in PTH to normalize serum calcium at the expense of bone [14–16]. Tolerance to the renal effects of caffeine does not develop with chronic use [17].

In a study in mice, it was found that caffeine effectively enhanced the osteoclastogenesis from bone marrow hematopoietic cells and bone resorption activity as assessed by the pit formation assay [18]. In another study, BMD was significantly lower in growing rats supplemented with 0.2% caffeine in diets for 20 weeks compared with the control group. Additionally, the calcium content in tibiae and femora of caffeine-treated rats was also lower, and the osteoclastogenesis of bone marrow cells isolated from caffeine-treated rats was markedly enhanced as compared with that in the control group. Taken together, these results suggest that caffeine reduces BMD through the enhancement of osteoclastogenesis and its calciuric effect [19].

Based on existing studies we hypothesize that caffeine usage, cumulative dose or duration of usage are associated with OP. and this association exists even when controlled for the effects of other neonatal risk factors.

The primary outcome of this study was to determine the effect of the cumulative dose and the duration of caffeine on OP. Other covariates of interest were included in the analysis, steroids and diuretics cumulative dose vitamin D intake, and maternal parity.

## Methods

This retrospective quantitative descriptive pilot cohort study was conducted at Health Sciences Centre in Winnipeg, Manitoba, Canada, from October 2007 to June 2012. Premature infants <31 weeks gestation and birth weight < 1500 g infants were included, all infants had at least 12 weeks of hospital stay. It is difficult to implement case control study having infants with no caffeine intake as all admitted infants less than 33 weeks are on caffeine by hospital guidelines. We excluded infants with congenital anomalies, infants with gut surgery affecting feeding, infants with non-osteopenic fractures, and infants with insufficient data to analyze. The data were collected from the charts in the medical record. The study included 109 infants who met the inclusion criteria. Cases of osteopenia were defined if they have radiological evidence of osteopenia of prematurity.

The data included: GA in weeks, gender, birth weight, average biweekly weight, total parenteral nutrition (TPN) days, and maternal parity level. The later was recorded as categorical data; high if >5, moderate if 3 or 4 and low parity if 1 or 2. Average biweekly vitamin D intake was included as longitudinal data. Serum phosphate measurements were collected on biweekly basis +/−1 week. The phosphate level was recorded as categorical data; high if >2.5 mmol/l, normal if between 1.8 to 2.5 mmol/l, low if between 1.3 to 1.8 mmol/l and very low if <1.3 mmol/l. The radiological data (X rays) were reviewed and interpreted, by a pediatric radiologist and the writer, (the Cohen’s kappa was 0.83 and 95% CI 0.82 to 0.084, which indicates very good interrater agreement) [20] both did not know the infants ‘clinical status or biochemical data at the time of the interpretation, on a biweekly basis at least for the first 12 weeks of life, using Koo et al. criteria [21]. Table 1.

**Table 1:** Koo et al. Criteria for osteopenia of prematurity

Grades	Description
Grade 0:	Normal density of bone cortex along shaft with normal dense white line at metaphysis and normal band of lucency, and thinning of cortex.
Grade 1:	Loss of dense white line at the metaphysis, increased sub-metaphyseal lucency and thinning of cortex.
Grade 2:	Changes in grade 1 plus irregularity and fraying of metaphysis, with splaying and cupping that is indicative of rickets.
Grade 3:	Indications of rickets with evidence of fractures.

The descriptive statistics (means and standard deviations) or (median and quartile) were used to summarize the characteristics of the sample. As the grade level of bone of newborn infants was measured fortnightly from birth to 12 weeks old, the binary outcome variables (OP) (0, 1), are longitudinal with up to 7 time points. It was preferable to include grade 1 and 2 of OP together, as the differentiation between the two grades is very subjective. Grade 3 OP was easier to distinguish, as callus formation was indicative of previous underlying spontaneous fracture. Due to the limited sample size, we dichotomized the radiological grading of OP by collapsing grades 1, 2 and 3 together as OP. At the same time, we considered grade 0 as normal. We assessed the OP status for every two weeks, Therefore, the generalized linear mixed model was used for repeated measures of binary outcome (OP status) [22].

The cumulative dose of caffeine were included in the generalized linear mixed model as covariates. Other covariates added to the generalized linear mixed model included doses of steroids, diuretics, vitamin D intake, and other demographic variables such as GA in weeks and gender. Vitamin D intake, average biweekly weight, and serum phosphate were treated as time-varying covariates. To examine whether the effect of duration of caffeine treatment on OP, a generalized linear mixed model was fitted by including the interaction between caffeine dosage and duration of therapy, and other covariates. The statistical analyses were carried out using SAS 9.3 (SAS Institute, Cary, NC). All *p*-values are two-sided, and significance was set at a value of 0.05.

## Results

The initial cohort included 335 preterm infants, with GA of less than 31 weeks and birth weight less than 1500 g, who were admitted to the NICU between July 2007 and July 2012. Of these 335 infants, 35 infants died, 5 infants were transferred to other facilities and 3 others who had surgical necrotizing enterocolitis with short bowel syndrome were also excluded. Out of the remaining 292 infants, the final study group included 109 infants who had the required 12 weeks of hospital stay, radiological data and laboratory data to analyze.

The raw data were examined for any outliers and influential points before the start of the analysis. The results of GA, birth weight, sex, maternal parity and (TPN) duration are shown in Table 2 as mean ± 2SD, and average biweekly weight and vitamin D intake in Table 3 as mean ± 2SD.

**Table 2:** The cohort biometric data

Variables	
Gestational Age (weeks) (mean ± 2SD)	27 ± 1.6
Birth Weight (grams) Mean ± 2SD	665 ± 229
Male/Female	54 male/55 female
Maternal Parity	
Low parity < 2	85 (77.9%)
Moderate parity2–4	16 (14.6%)
High parity > 4	8(7.5%
TPN days	
(Median)	21
Quantiles	11, 32

**Table 3:** The average biweekly weight and vitamin D intake of the study cohort

	Week1–2	Week3–4	Week5–6	Week7–8	Week9–10	Week11–12
Average weight in grams (mean ± 2SD)	993 ± 23	1108 ± 2	1335 ± 29	1660 ± 4	1984 ± 4	2348 ± 5
Average Vitamin D in units (mean ± 2SD)	392 ± 35	555 ± 37	737 ± 33	834 ± 29	947 ± 29	1034 ± 32

There were 8 infants with bone fractures (8%). The fractures involved the right and left lower ribs and none of them had a spontaneous fracture of the humerus. The prevalence of OP based on Koo et al. in this cohort was 51.3%.

All the infants received caffeine during their hospital stay, starting day one. The mean ± 2SD dose of caffeine was 425.33 ± 235.2 mg as a cumulative dose and the mean ± 2SD duration of caffeine therapy was 60 ± 45.8 days. The mean ± 2SD dose of caffeine was 7.95 ± 2.7 mg per kg per day and the range of caffeine dose was (4.1–15.6 mg/kg/day) including the loading, the maintenance dose and the mini-load doses. The usual starting load was 10 mg/kg followed by maintenance of 5–7 mg/kg/day and the infant received mini-loads of caffeine in-between according to the severity of apnea of prematurity as long as the heart rate was less than 180 beat/min. During the study time, there was no systematic protocol to monitor the serum caffeine level.

There were 79 infants who received diuretics (73%). The median diuretic dose was 5.9 mg with 1st and 3rd quartiles of 1, 25.8 during the hospital stay. The steroids were calculated as dexamethasone dose or equivalent as 100 mg of hydrocortisone are equal to 20 mg of dexamethasone. In this cohort, the median steroid dose was 2 mg and the 1st and 3rd quartiles were 0, 42 mg during the hospital stay.

We first fitted a logistic regression model to examine each individual variable associated with the probability of OP, including gestational age, average biweekly birth weight, maternal parity, TPN duration, vitamin D intake, and serum phosphate level, duration of caffeine treatment and the cumulative doses of caffeine, steroids, and diuretics. The results are presented in Table 4. Table 4 shows that lower gestational age and average biweekly weight are correlated with OP. Similarly, higher caffeine cumulative dose and longer caffeine duration of therapy showed a statistically significant correlation with OP (p* < 0.05). In the univariate model; steroids doses, TPN days and average biweekly intake of vitamin D displayed significant correlation with OP. On the contrary, maternal parity, serum phosphate and diuretics were not associated with OP (*p* > 0.05) in this study. The maternal parity was analyzed as low parity if less than 2 and moderate parity if more than 2. Similarly, serum phosphate was categorized as very low if less than 1.3 mmol/l and low if between 1.3 and 1.8 mmol/l and normal if more than 1.8 mmol/l.

**Table 4:** Factors associated with OP: Results of univariate analysis

Variables	Estimate	Standard Error	*P* value
Gestational age (weeks)	−0.645	0.147	<0.001*
Average biweekly weight (grams)	0.0006	0.0002	0.006*
Caffeine cumulative dose (mg)	0.005	0.001	<0.001*
Caffeine duration (days)	0.051	0.013	<0.001*
Steroids cumulative dose (mg)	0.09	0.046	0.038
TPN duration (days)	0.034	0.012	0.005*
Vitamin D (units)	−1.863	0.36	<0.001*
Diuretics cumulative dose (mg)	0.003	0.002	0.20
Serum phosphate (mmol/l)			
Phosphate <1.3	−0.09	0.16	0.57
Phosphate (1.3–1.8)	0.11	0.33	0.74
Phosphate >1.8 (ref)			
Maternal Parity			
Low parity	−0.016	0.42	0.96
Moderate Parity (ref)			

* Means significant

Then we fitted a logistic multivariable generalized linear mixed model with gestational age, average biweekly weight, cumulative dose of caffeine, cumulative steroids dose and vitamin D considering the clinical importance and statistical significance at univariate analysis. The results are showed in Table 5.

**Table 5:** Results from Multivariable generalized linear mixed model

Effect	Estimate (logit)	Standard Error	*P* value
Intercept	5.63	5.59	0.321
Caffeine Cumulative Dose (mg)	0.39	0.05	0.007*
Steroid Cumulative Dose (mg)	0.17	0.05	0.035*
Vitamin D (units)	−1.64	0.47	0.006*
Average Biweekly Weight (grams)	−0.01	0.0001	<0.0001*
Gestational age (weeks)	−0.41	0.19	0.0408*

p* = significant value

Table 5 indicates that higher cumulative dose of caffeine is associated with an increase in the probability of OP. The effect of caffeine was true even when we controlled the effect of other variables (average weight, the gestational age, steroid and vitamin D). The odds of OP is 1.10 times (95%CI: 1.05–1.15) higher for every 5 mg/kg increase in cumulative caffeine dose when other factors are controlled.

The steroid dosage has a statistically significant result in predicting OP with (p* < 0.0001) (estimated Odds ratio = 1.1 and CI: 1.005–1.20).

The results showed that the average biweekly vitamin D intake, both included in the diet and supplemented, had a negative correlation with the OP (p* < 0.0001). The probability of OP is decreased by 0.4% when vitamin D increased from 400 to 800 units.

**Fig. 1:**
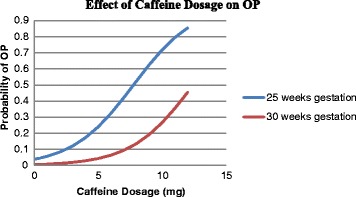
Probability of OP with increasing caffeine dosage at 25 weeks and at 30 weeks gestational age based on the logistic model

Figure 1 shows the effect of increasing caffeine dosage on the probability of OP over time in different gestational age (25 weeks GA = 15 infants and 30 weeks GA = 25 infants) based on the above fitted logistic generalized linear mixed model.

To examine whether the effect of duration of caffeine treatment, we fitted another generalized linear mixed model by including the interaction between caffeine dosage and duration of therapy, and other covariates, the results are showed in Table 6. This table shows that, the average caffeine dose, caffeine duration of therapy as well as the interaction between caffeine dose and duration of caffeine treatment has a statistical significant correlation with OP even when controlling for the effects of gestational age, weight and vitamin D (*p* < 0.05).

**Table 6:** Estimates with interaction of caffeine and duration of treatment

Effect	Estimate (logit)	Standard Error	P
Intercept	3.39	5.99	0.57
Average Caffeine dose (mg/kg/d)	0.24	0.09	0.029*
Duration of caffeine treatment (days)	0.64	0.27	0.02*
Caffeine dose* Duration of caffeine treatment (days)	0.07	0.04	0.05*
Steroid cumulative dose (mg)	0.09	0.05	0.07
Vitamin D (units)	−1.86	0.36	0.04*
Average biweekly Birth Weight (grams)	−0.06	0.02	0.001*
Gestational age (weeks)	−0.64	0.15	0.001*

p* Indicates significant level

**Fig. 2:**
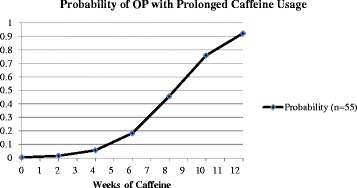
Probability of OP with prolonged caffeine use based on the logistic model

**Fig. 3:**
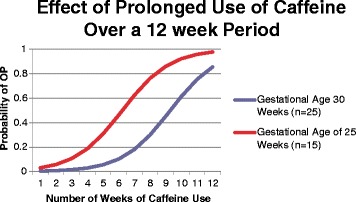
Probability of OP with same Caffeine dosage at 25 weeks and 30 weeks gestational age over the weeks of treatment based on the logistic model

Based on the model in Table 6, Figs. 2 and 3 show the effect of duration of caffeine usage on the probability of OP based on the logistic model. The probability of OP increased in 25 weeks preterm infants (15 infants), is higher than the 30 weeks preterm infants (25 infants). The figure exhibited that the lower the gestational age the higher the probability of osteopenia over prolonged caffeine use, even when controlling caffeine dose, steroid dose, birth weight, and vitamin D.

## Discussion

Although the overall survival of extreme low birth weight infants has improved over the past 2 decades, these infants continue to have significant comorbidities. The prevalence of OP in our study is similar to that previously reported in the literature and suggests that OP remains a significant comorbidity in extreme low birth weight infants and puts them at increased risk for spontaneous fractures during the NICU stay. Our results are consistent with this concept, the younger and smaller the babies, the higher the incidence of OP.

The results of this study revealed a strong correlation between caffeine treatment and the presence of OP. Despite caffeine’s effect on treating apnea of prematurity with favorable long-term outcomes [23], our study revealed a strong association between cumulative dosage and duration of treatment with caffeine and OP even when controlling for the effect of other risk factors. The results show that the adverse effect of caffeine is more evident in lower gestational age infants, which may be explained by the prolonged half-life of caffeine in their bodies due to diminished kidney abilities to eliminate the caffeine. Furthermore, extreme preterm infants have immature liver enzymes and are unable to catabolize caffeine leading to a prolonged effect causing calciuria and osteoclastogenesis [14, 19].

In contrast to the current study results, a retrospective study done by Viswanathan et al. (2014), showed that there was no difference in duration of caffeine use between cases of OP and the control group. Viswanathan et al. did not calculate caffeine dose, only caffeine duration was tracked between cases and controls. Additionally, in the Viswanathan et al. (2014) study, infants with spontaneous rib fractures were included in the control group if there was no radiological evidence of OP. In our study, the osteopenic fractures were encompassed in the cohort data and identified as having severe grade osteopenia. The average duration of caffeine treatment in both groups in the Viswanathan et al. study was 40 days, while in our study, the average duration of caffeine treatment was 60 days [24].

Our current study was a retrospective one and there was no accurate documentation of maternal caffeine intake during pregnancy and lactation time. However, it is worth mentioning that in an animal study, maternal caffeine intake negatively affected bone formation and development [25]. Thus our results may still imply an effect of maternal caffeine exposure either in utero or through mother’s breast milk and donor breast milk. However, the high doses of caffeine prescribed for apnea of prematurity have paramount contribution to OP.

In this study, there was no difference between male and female infants regarding OP, which is in agreement with another comparable study [26]. But our results do differ from other published studies which found that male infants have higher bone density than females when comparing preterm male and female infants with male and female full term newborns. Such an observation may follow a recognizable trend for testosterone hormone in utero [27, 28].

This study showed significant effect of TPN duration on the development of OP but this effect disappeared when we controlled for other risk factors. This can be explained by considering that other factors contribute more to OP, and that TPN contains the maximum amount of calcium and phosphate according to the maximum solubility allowed [29]. In this study, TPN duration count included the null per os days as well as partial feeding days. During the study time, TPN is provided till the infant can tolerate the full enteral feeding.

Although Backström et al. suggested that serum phosphate levels lower than 1.8 mmol/L (5.5 mg/dl) may have a diagnostic sensitivity of 100% and specificity of 70% for OP [30], in our study, serum phosphate on biweekly basis did not show a statistically significant correlation with OP. No other published studies have examined serum phosphate as a longitudinal marker over the hospital stay. Yet, serum phosphate is among the minerals that are regulated tightly, and the average biweekly record may not represent the real situation of serum phosphate in infants on TPN for the first week at least and partial feeding for another week. In agreement with our results, Aly et al., (2005) found that serum phosphate as a single reading at birth was not correlated with OP in preterm infants [27]. In another study serum phosphate and serum alkaline phosphatase were correlated with OP later in infancy, which could be explained by the other confounding factors and medications received that affect premature bone in early life in NICUs [31, 32].

While it is documented that the number of previous pregnancies of a healthy mother correlated negatively with BMD measurements, the effect of previous pregnancies did not show the same effect on infants’ bone formation. This supports the fact that an infant acquires the needed minerals and vitamin from the mother’s body with active transport against the concentration gradient ignoring the mother’s general status [33]. In our study, there was no significant effect of maternal parity on OP. On the other hand, this cohort study with limited sample size did not have enough high parity mothers to detect a correlation, and thus further research is needed that includes high parity mothers.

Our study results show a statistically significant correlation between OP and steroid cumulative dose, while diuretics did show a positive trend in relation to OP. This correlation did not reach statistical significance. This result can be explained by the short duration of diuretics use and the relative small sample size. The use of high dose of caffeine that has a diuretic effect might explain the lower need for the diuretic use.

## Conclusions

We conclude that caffeine has a strong association with OP. As limit of viability continues to decrease with 70% survival of infants between 24 and 26 weeks, OP will continue to increase and will results in significant morbidity in childhood and adulthood unless strategies to mitigate risk factors are developed. Our study was limited by the small sample size. The study was conducted at one center, and thus the results may not be generalizable on a wider scale. Further studies are needed to determine effective lower caffeine dosage, different ventilation strategies, adequate vitamin D intake, and passive movement as all these can provide protection against OP.

## Abbreviations

BMC: Bone mineral content
BMD: Bone mineral density
BW: Birth weight
GA: Gestational age
NICU: Neonatal intensive care unit
OP: Osteopenia of prematurity
PTH: Parathyroid hormone
TPN: Total parenteral nutrition
